# Tau Protein Modulates Perineuronal Extracellular Matrix Expression in the TauP301L-*acan* Mouse Model

**DOI:** 10.3390/biom12040505

**Published:** 2022-03-26

**Authors:** Sophie Schmidt, Max Holzer, Thomas Arendt, Mandy Sonntag, Markus Morawski

**Affiliations:** Paul Flechsig Institute of Brain Research, Medical Faculty, University of Leipzig, 04103 Leipzig, Germany; sophie.schmidt@medizin.uni-leipzig.de (S.S.); max.holzer@medizin.uni-leipzig.de (M.H.); thomas.arendt@medizin.uni-leipzig.de (T.A.); mandy.sonntag@medizin.uni-leipzig.de (M.S.)

**Keywords:** perineuronal nets, aggrecan, tau pathology, neuroprotection, P301L, sulfated proteoglycans

## Abstract

Tau mutations promote the formation of tau oligomers and filaments, which are neuropathological signs of several tau-associated dementias. Types of neurons in the CNS are spared of tau pathology and are surrounded by a specialized form of extracellular matrix; called perineuronal nets (PNs). Aggrecan, the major PN proteoglycans, is suggested to mediate PNs neuroprotective function by forming an external shield preventing the internalization of misfolded tau. We recently demonstrated a correlation between aggrecan amount and the expression and phosphorylation of tau in a TauP310L-*acan* mouse model, generated by crossbreeding heterozygous aggrecan mice with a significant reduction of aggrecan and homozygous TauP301L mice. Neurodegenerative processes have been associated with changes of PN structure and protein signature. In this study, we hypothesized that the structure and protein expression of PNs in this TauP310L-*acan* mouse is regulated by tau. Immunohistochemical and biochemical analyses demonstrate that protein levels of PN components differ between TauP301L^HET^-*acan*^WT^ and TauP301L^HET^-*acan*^HET^ mice, accompanied by changes in the expression of protein phosphatase 2 A. In addition, tau can modulate PN components such as brevican. Co-immunoprecipitation experiments revealed a physical connection between PN components and tau. These data demonstrate a complex, mutual interrelation of tau and the proteoglycans of the PN.

## 1. Introduction

Tau protein is a neuron-specific cytoskeletal protein in the central nervous system (CNS) and peripheral nervous system [1]. It is primarily linked to axonal microtubules [2,3] but is also present at low levels in dendrites [4,5,6]. Axonal located tau protein is responsible for the assembly and stabilization of microtubules while dendritically located tau is involved in signaling functions [7]. Tau protein is subjected to numerous post-translational modifications [8,9] and interacts with various proteins [7,10,11,12,13]. Alterations in tau protein modification such as phosphorylation and expression cause relocation of tau from neuronal processes to the somata, which coincides with the formation of harmful oligomers and aggregates [14,15]. Such formation of toxic tau accumulations is an essential neuropathological hallmark of tau-associated dementias like Alzheimers disease (AD), frontotemporal dementia, and parkinsonism linked to chromosome 17 (FTDP-17) and Picks disease [16,17,18]. The progression of misfolded tau protein seems to follow a predictable manner along connected anatomical pathways, that is, however, specific for each form of dementia [19,20,21]. Thus, the distribution of pathological features is restricted to certain brain regions or even types of neurons [19,20,21]. Brückner and colleagues [22] first showed the protective function of a specialized subtype of the neuronal extracellular matrix (ECM) called perineuronal nets (PNs) against neuronal tau aggregation. PNs surround only specific subgroups of neurons throughout the brain and are mainly composed of chondroitin sulfated proteoglycans (CSPGs) of the lectican family (such as aggrecan, brevican, neurocan, and versican), which form a quaternary complex by an interaction with hyaluronic acid (HA), hyaluronan and proteoglycan link proteins (HAPLNs) and tenascins, mainly tenascin-R [23,24,25,26,27,28,29,30,31,32,33,34]. Investigation of *post-mortem* brains of AD patients revealed that areas with a high-density of PN-associated neurons are less severely affected by tau pathology compared to areas with sparse number of PN-positive neurons. Furthermore, PN-associated neurons appear to be virtually devoid of neurofibrillary tangles (NFTs) even in advanced stages of AD [22,35,36,37], leading to the assumption that PNs serve neuroprotective functions. In a series of follow-up studies the CSPG aggrecan was identified to be the carrier of this function [38,39]. However, the biological basis for the selective vulnerability of neurons in tauopathies and the relation to neuroprotection by aggrecan remains elusive.

In a previous work, we generated the TauP301L-*acan* mouse [40] by crossbreeding TauP301L mice [41,42] and heterozygous aggrecan mice [43,44,45] with the aim to gain more knowledge on the mechanisms of the neuroprotective action of aggrecan against tau induced pathology. All offsprings revealed one allele of murine tau and one allele of P301L human tau and either one or two alleles of functional aggrecan. The deficit of one functional aggrecan allele in the heterozygous aggrecan genotype was linked to a ~60% reduction of aggrecan protein level [40]. Though both genotypes of this mouse were genetically identical with respect to the P301L mutation of tau and murine tau, we surprisingly detected that the total protein amount of transgenic human P301L tau as well as endogenous murine tau was significantly increased in the heterozygous aggrecan genotype compared to the wildtype aggrecan genotype in this mouse model, confirming a regulatory effect of aggrecan on tau expression and indicating a more severe tauopathy when aggrecan is reduced [40].

Interestingly, tauopathies as well as Aβ-pathologies have been linked to significant changes in general PN structure and protein expression [46,47]. Further, sulfated proteoglycans were shown to bind to tau protein and directly influence tau spreading, uptake, and aggregation [48,49]. These findings might also indicate a mutual interaction of tau and proteoglycans of PNs and raise the assumption that tau protein might have the potential to directly control the formation and mature structure of perineuronal nets. In the present study, we addressed this hypothesis by investigating the structure and protein expression of PNs in mice with different levels of tau expression. As pathological tau species propagate extracellularly and are supposed to spread trans-synaptically through anatomically connected networks [50,51], we further investigated the potential of PN components to physically interact with tau.

## 2. Materials and Methods

### 2.1. Animals

All experiments were carried out in (1) wildtype aggrecan mice with two functional aggrecan alleles (strain: *Acan^cmd^*/NKruJ mice; further termed *acan*^WT^) [43,44], (2) tau knockout mice (strain: *Mapt^tm1(EGFP)Klt^/J* mice, further termed *Mapt* ^KO^) [42] and (3) TauP301L-*acan* mice with either two or one functional aggrecan alleles (TauP301L-*acan*^WT^ and TauP301L-*acan*^HET^) and in a hybrid background (BALB/c and C57BL/6J) [40]. TauP301L-*acan* mice [40] were generated by crossbreeding of heterozygous *aggrecan* mice (strain: *Acan^cmd^*/NKruJ mice), which are characterized by a spontaneous deletion of 7 bp within the coding region of one aggrecan allele [43] resulting in a significant reduction of aggrecan protein amount [52] with homozygous TauP301L mice on a murine tau null background [40] (crossbreds of JNPL3(P301L) mice [41] × *Mapt* ^KO^ mice [42]), which overexpress human P301L mutated tau protein (4R/0N isoform) and built up tau aggregates in brain stem and thalamus. BALB/c mice and C57BL/6J mice were used to identify strain-specific differences. The genotype of the experimental animals was determined by PCR. The animals were bred in the animal care facility of the Medical Experimental Center of the Faculty of Medicine of the University of Leipzig in a temperature-controlled environment with free access to food and water and 12-h dark/light cycle. Experiments were performed on female mice [40].

### 2.2. Immunohistochemistry

Immunohistochemistry was performed in 10–11-month-old female mice [40]. Animals (3 TauP301L-*acan*^WT^; 3 TauP301L-*acan*^HET^) were euthanized with CO_2_ and perfused transcardially with 0.9% NaCl containing 5 I.E./mL heparin followed by fixative (4% paraformaldehyde and 0.1% glutaraldehyde). Brains were removed from skull, postfixed overnight, cryo-protected in 30% sucrose containing 0.1% Na-azide, and sectioned in 30 µm coronal slices with a cryomicrotome (Zeiss Hyrax S30 with freezing unit Zeiss Hyrax KS34, Jena, Germany). Before staining, brain sections were treated with 60% methanol and 2% H_2_O_2_ for 30 min, followed by blocking solution (2% bovine serum albumin (BSA), 0.3% milk powder and 0.5% donkey serum) for 1 hr. Primary antibodies (Table 1) were diluted in blocking solution and incubated for 2 d at 4 °C. Visualization of immunoreactivity was executed by using fluorochrome-conjugated secondary antibodies.

Fluorescence microscopic analysis was performed by using a Zeiss confocal laser scanning microscope LSM 880 Airyscan (Carl Zeiss, Jena, Germany). Images were obtained using a 20× (0.8 numerical aperture) objective and a 63× water immersion (1.2 numerical aperture) objective. Fluorescence excitation was applied with HeNe lasers (563 nm, Cy3; 633 nm, Cy5) and the emission was detected using band-pass filters: 565–615 nm for Cy3 and 650–710 nm for Cy5. Z-stacks ranging ~15 µm were taken at distances of 410 nm (number of z stacks: ~37 slices) for 20× objective and 240 nm (number of z stacks: ~63 slices) for 63× objective. Zen 2.3 software (Carl Zeiss, Göttingen, Germany) was used to process the images.

Fluorescence images were processed by identical settings and adjustments to ensure comparable conditions for image analysis. Intensity and location of immunoreactions were estimated by semiquantitative analysis. High magnification confocal z-stack images in orthogonal view were used to distinguish intra- and extracellular fluorescence signals as well as the intracellular colocalization of PN molecules and tau. An extracellular location was defined by a specific immunoreaction delineating an unlabeled, central area (≥10 µm diameter) containing the nucleus (visualized with Hoechst 33342) while an intracellular location was characterized as an immunoreaction tightly surrounding the nucleus.

### 2.3. Tissue Extraction for Biochemical Analysis

Animals (Western blot and ELISA: 8 TauP301L^HET^-*acan*^WT^, 8 TauP301L^HET^-*acan*^HET^, 3 *acan*^WT^, 4 *Mapt ^KO^*, 5 BALB/c mice, 5 C57BL/6J; Immunoprecipitation: 3 C57BL/6J, 1 *Mapt ^KO^*) were euthanized by cervical dislocation, brains were immediately removed, and a slice of Mo5-bearing brain tissue (Bregma −4.8 to Bregma −5.5) was shock frozen in liquid nitrogen. Brain tissue was extracted with 10× volume of ice-cold homogenization buffer (20 mM Tris, 150 mM NaCl, 0,5% Triton X-100, 2 mM EDTA) containing cOmplete^TM^ protease inhibitor cocktail (Sigma-Aldrich, Taufkirchen, Germany) and PhosSTOP (Sigma-Aldrich, Taufkirchen, Germany) using a bead-based homogenizer (Precellys^®^ 24, Bertin-Technologies, Rockville, MD, USA). The homogenates were centrifuged at 10.000× *g* for 10 min at 4 °C, the supernatants were recovered, and total protein concentration was determined using BCA kit (Pierce, Rockford, IL, USA).

### 2.4. Western Blot

Equal amounts of protein extracts were separated under reducing and denaturating conditions on SDS-PAGE and transferred to a polyvinylidene difluoride membrane (GE Healthcare, Freiburg, Germany) overnight at 25 V. Blots were incubated with blocking solution (1% BSA in TBS-T (0.05% Tween 20)) for 1 h and probed overnight at 4 °C with the primary antibody diluted in blocking solution (Table 1). Blots were washed 3 times with TBS-T followed by incubation with appropriate peroxidase-conjugated secondary antibodies for 1 h.

HRP activity was visualized by means of chemoluminescence reagent (Lumigen ECL Ultra, Lumigen Inc., Southfield, MI, USA) and imaged with a chemoluminescence detection system (Fusion FX, Vilber, Lourmat, Eberhardzell, Germany).

Quantification was performed on original data files. Ratios of optical density were determined by means of TotalLab v2005 (Nonlinear Dynamics, Newcastle upon Tyne, UK) and normalized to total protein content of each lane stained by Coomassie R250 [60].

### 2.5. Enzyme-Linked Immunosorbent Assay (ELISA)

For the detection of HA, high binding microplates (Greiner Bio-One GmbH, Frickenhausen, Germany) were coated overnight at 4 °C with 50 µL brain homogenates (5 mg/mL) 1:4000 diluted in carbonate buffer (0.1 M NaCO_3_, pH 9.7). Coated plates were washed with TBS-T (0.05% Tween 20) and incubated with blocking solution (1% BSA in TBS-T) for 1 h and probed with biotinylated hyaluronic acid binding protein (Merck Millipore, Darmstadt, Germany, RRID: AB_2861303) at a concentration of 0.125 µg/mL for 1 h. After washing, the plates were incubated with ExtrAvidin^®^-Peroxidase (Sigma-Aldrich, Taufkirchen, Germany) for 45 min and developed by using 100 µL/well 3,3′,5,5′-tetramethylbenzidine solution. The reaction was stopped after 25 min with 1 N H_2_SO_4_ and absorbance was measured at 450 nm (microplate reader Mithras LB 940, Berthold Technologies, Bad Wildbad, Germany).

### 2.6. Co-Immunoprecipitation

For the immunoprecipitation Dynabeads^TM^ Protein A and G immunoprecipitation kit (ThermoFisher Scientific, Waltham, MA, USA) was used according to the manufacturer’s protocol. Antibodies (Table 1) were diluted in antibody binding and washing buffer and incubated with 50 µL Dynabeads for 3 h at room temperature with rotation to generate Dynabeads-antibody complex. After washing, 500 µg brain homogenate was added to each Dynabead-antibody complex and incubated overnight at 4 °C with rotation. The Dynabead-antibody-antigen complex was washed 3 times with washing buffer, eluted with 20 µL elution buffer and analyzed by means of Western blot (described above). The antibodies are summarized in (Table 1).

### 2.7. Statistics

Statistical analysis was performed in SigmaPlot 13 (Systat Software, Erkrath, Germany). Comparison of two genotypes was conducted by Students *t*-test and data are provided as mean ± SD.

## 3. Results

### 3.1. Characterization of Perineuronal Nets in Brains of TauP301L-acan Mice

As the proper function of PNs is based on an interplay of its individual components [34,39,61,62,63], we initially characterized the appearance and amount of major PN components in the two aggrecan genotypes of the TauP301L-*acan* mouse. Recent studies showed that especially older female mice exhibited pronounced pathological changes in the brainstem, while males were mostly spared of any pathology [40,64]. Hence, the subsequent analyses were performed exclusively on female mice. The biochemical investigations were carried out in the brainstem, which is particularly highly enriched in aggrecan [54,65]. The immunohistochemical analyzes were performed in the trigeminal motor nucleus (Mo5), which mainly consists of cholinergic motor neurons [66,67]. Neurons of the cholinergic transmitter system are known to be susceptible to tau pathology [68,69].

#### 3.1.1. Brevican

**Figure 1:**
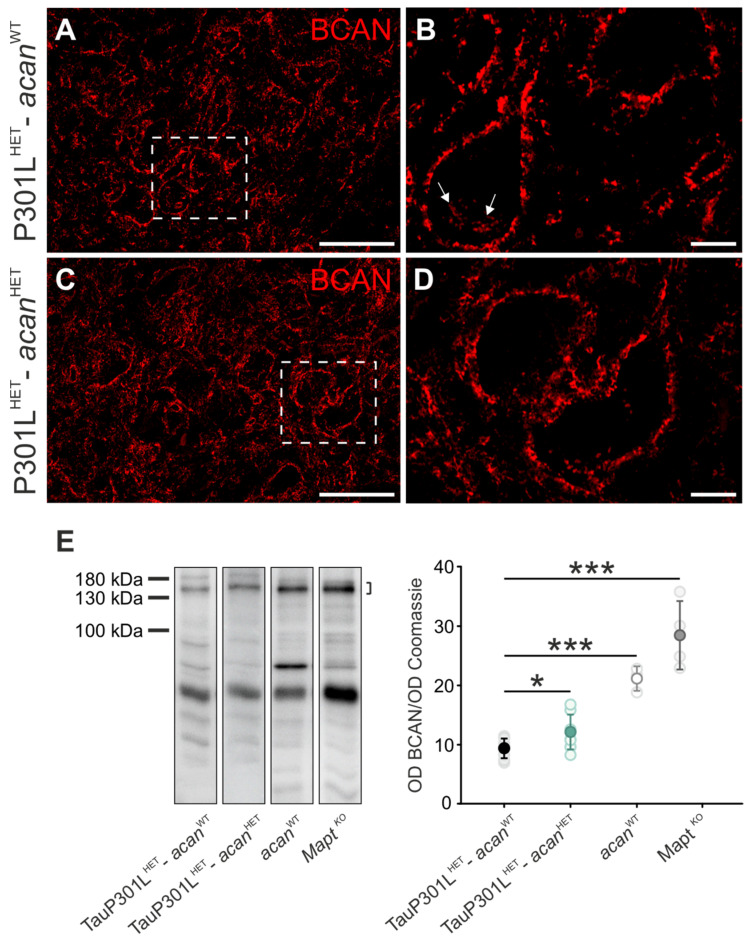
Characterization of brevican in the Mo5 of TauP301L-*acan* mice. (**A**–**D**) Immunohistochemical analysis of brevican (BCAN, clone 2 (BD Biosciences) in the motor trigeminal nucleus (Mo5) of 3 TauP301L^HET^-*acan*^WT^ (**A**,**B**) and 3 TauP301L^HET^-*acan*^HET^ mice (**C**,**D**). High magnification confocal images (**B**,**D**) of brevican immunoreaction visualize location and structure of the immunoreaction. Brevican was mostly localized extracellularly in TauP301L^HET^-*acan*^HET^ (**C**) and TauP301L^HET^-*acan*^WT^ mice (**A**). The structure of brevican immunoreaction did not yield any genotype-specific differences. However, an intracellularly clustered brevican immunoreactivity was detected in a few cells of TauP301L^HET^-*acan*^WT^ mice ((**B**), arrows). (**E**) Western blot analysis of the amount of brevican full length protein (clone 2 (BD Biosciences), at ~145 kDa) in the brainstem with a significant increase in TauP301L^HET^-*acan*^HET^ mice compared to TauP301L^HET^-*acan*^WT^ mice (*p* = 0.035, *n* = 8, *t*-test). Comparison of brevican protein level in the control mice yielded striking differences. Brevican protein level was significantly reduced in TauP301L^HET^-*acan*^WT^ mice compared to *acan*^WT^ mice (*p* < 0.001, TauP301L^HET^-*acan*^WT^ *n* = 8, *acan*^WT^ *n* = 3, *t*-test). The protein amount of brevican full length protein was significantly increased in *Mapt ^KO^* mice compared to TauP301L^HET^-*acan*^WT^ mice (*p* < 0.001, *Mapt ^KO^ n* = 4, TauP301L^HET^-*acan*^WT^ *n* = 8, *t*-test). For Western blot analysis protein extracts from homogenized brainstems of 8 TauP301L^HET^-*acan*^WT^, 8 TauP301L^HET^-*acan*^HET^, 3 *acan*^WT^ and 4 *Mapt^KO^* mice were used. Optical density (OD) of the specific chemoluminescence signal was quantified in relation to Coomassie stained total protein bands. Quantified areas on the Western blot lanes were marked by a bracket. For illustration of exemplary blot lanes, brightness and contrast were adapted. Data are given as mean ± SD,* *p* < 0.05, *** *p* < 0.001. Scale bar in (**A**,**C**) 50 µm, in (**A**,**D**) 10 µm.

Immunohistochemical analysis of brevican revealed a prominent, mostly extracellular immunoreactivity in TauP301L^HET^-*acan*^WT^ (Figure 1A,B) and TauP301L^HET^-*acan*^HET^ mice (Figure 1C,D) which surrounds the large, cholinergic Mo5 motor neurons. However, brevican immunoreaction was also detected intracellularly in some cells of TauP301L^HET^-*acan*^WT^ mice (Figure 1B, arrows). The intracellular immunoreaction exhibited a clustered appearance.

Subsequently, the brevican protein level was investigated by means of Western blot analysis (Figure 1E). The analysis of brainstem homogenates revealed a significantly increased brevican protein level in TauP301L^HET^-*acan*^HET^ (12.18 ± 2.93, *n* = 8) mice compared to TauP301L^HET^-*acan*^WT^ mice (9.35 ± 1.65, *p* = 0.035, *n* = 8, *t*-test, Figure 1E).

In comparison to TauP301L^HET^-*acan*^WT^ mice brevican protein level was significantly increased in *acan*^WT^ mice (TauP301L^HET^-*acan*^WT^: 9.35 ± 1.65, *n* = 8; *acan*^WT^: 21.13 ± 2.06, *n* = 3, *p* < 0.001, *t*-test, Figure 1E) and *Mapt ^KO^* mice (TauP301L^HET^-*acan*^WT^: 9.35 ± 1.65, *n* = 8; *Mapt ^KO^* 28.43 ± 5.78, *n* = 4, *p* < 0.001, *t*-test, Figure 1E). Comparison of brevican protein amount in C57BL/6 and BALB/c mice did not yield any genotype-specific difference (data not shown). Therefore, it can be excluded that strain-specific differences of the background strains are the cause of the significantly increased protein level of brevican in *acan*^WT^ and *Mapt ^KO^* compared to TauP301L^HET^-*acan*^WT^ mice.

#### 3.1.2. Neurocan

**Figure 2:**
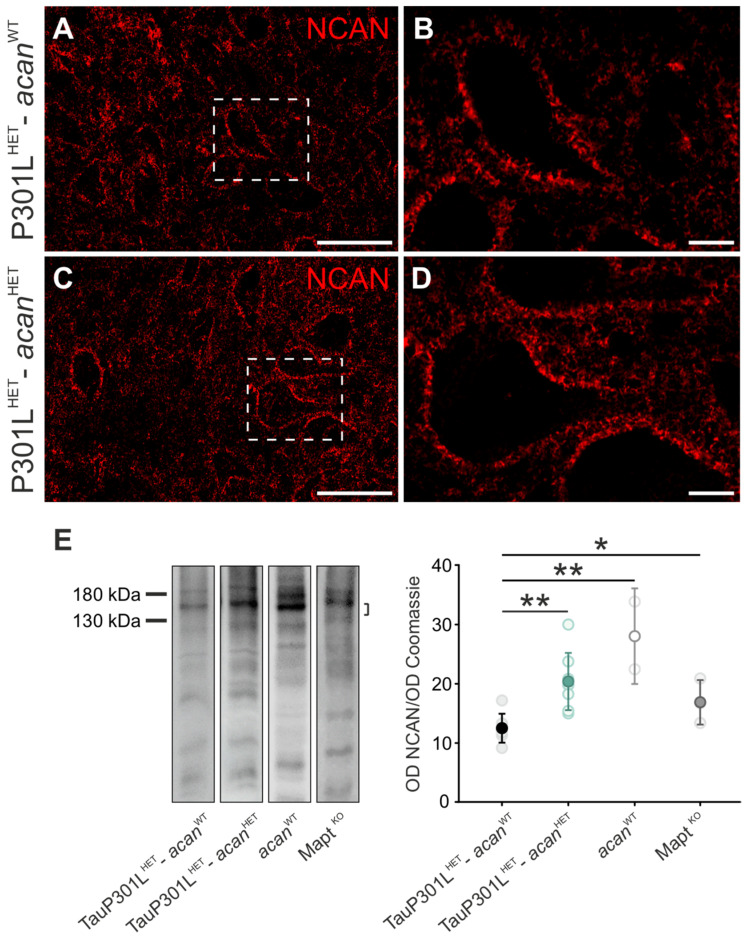
Characterization of neurocan in the Mo5 of TauP301L-*acan* mice. (**A**–**D**) Immunohistochemical analysis of neurocan (NCAN, AF5800 (R&D Systems)) in the motor trigeminal nucleus (Mo5) of 3 TauP301L^HET^-*acan*^WT^ (**A**,**B**) and 3 TauP301L^HET^-*acan*^HET^ mice (**C**,**D**). High magnification confocal images (**B**,**D**) of neurocan immunoreaction visualize location and structure of the immunoreaction. Location and structure of the neurocan immunoreaction did not reveal any genotype-specific differences (**A**,**C**). Neurocan immunoreaction was exclusively detected extracellularly (**B**,**D**). (**E**) Western blot analysis of neurocan protein level (AF5800 (R&D Systems), at ~150 kDa) in the brainstem with a significant increase in TauP301L^HET^-*acan*^HET^ mice compared to TauP301L^HET^-*acan*^WT^ mice (*p* = 0.001, TauP301L^HET^-*acan*^WT^ *n* = 7, TauP301L^HET^-*acan*^HET^ *n* = 8, *t*-test). Neurocan protein level was significantly increased in *acan*^WT^ mice compared to TauP301L^HET^-*acan*^WT^ mice (*p* = 0.001, TauP301L^HET^-*acan*^WT^ *n* = 7, *acan*^WT^ *n* = 2, *t*-test). *Mapt ^KO^* mice revealed a significantly increased neurocan protein amount compared to TauP301L^HET^-*acan*^WT^ mice (*p* = 0.049, TauP301L^HET^-*acan*^WT^ *n* = 7, *Mapt ^KO^ n* = 3, *t*-test). For Western blot analysis protein extracts from homogenized brainstems of 7 TauP301L^HET^-*acan*^WT^, 8 TauP301L^HET^-*acan*^HET^, 2 *acan*^WT^, and 3 *Mapt ^KO^* mice were used. Optical density (OD) of the specific chemoluminescence signal was quantified in relation to Coomassie stained total protein bands. Quantified areas on the Western blot lanes were marked by a bracket. For illustration of exemplary blot lanes, brightness and contrast were adapted. Data are given as mean ± SD, * *p* < 0.05, ** *p* < 0.01. Scale bar in (**A**,**C**) 50 µm, in (**B**,**D**) 10 µm.

Immunohistochemical analysis of the CSPG neurocan in TauP301L^HET^-*acan*^WT^ (Figure 2A,B) and TauP301L^HET^-*acan*^HET^ mice (Figure 2C,D) did not reveal any structural genotype-specific differences. Neurocan immunoreaction was detected extracellularly around cholinergic Mo5 motor neurons and in the neuropil (Figure 2B,D).

Furthermore, Western blot analyses were performed to investigate the neurocan protein level. TauP301L^HET^-*acan*^HET^ mice (20.31 ± 4.83, *n* = 8) revealed a significantly increased neurocan protein level compared to TauP301L^HET^-*acan*^WT^ mice (12.43 ± 2.43, *p* = 0.001, *n* = 7, *t*-test, Figure 2E).

Neurocan protein level was significantly increased in *acan*^WT^ mice (28.09 ± 8.07, *n* = 2) compared to TauP301L^HET^-*acan*^WT^ mice (12.43 ± 2.43, *p* = 0.001, *n* = 7, *t*-test, Figure 2E). *Mapt ^KO^* mice (16.93 ± 3.76, *n* = 3) also revealed a significantly increased protein level of neurocan compared to TauP301L^HET^-*acan*^WT^ mice (12.43 ± 2.43, *p* = 0.049, *n* = 7, *t*-test, Figure 2E). The possibility that these striking differences are based on strain-specific differences again can be excluded as the analysis of neurocan protein level in BALB/c and C57BL/6 mice did not yield any difference (data not shown).

#### 3.1.3. HAPLN1

**Figure 3:**
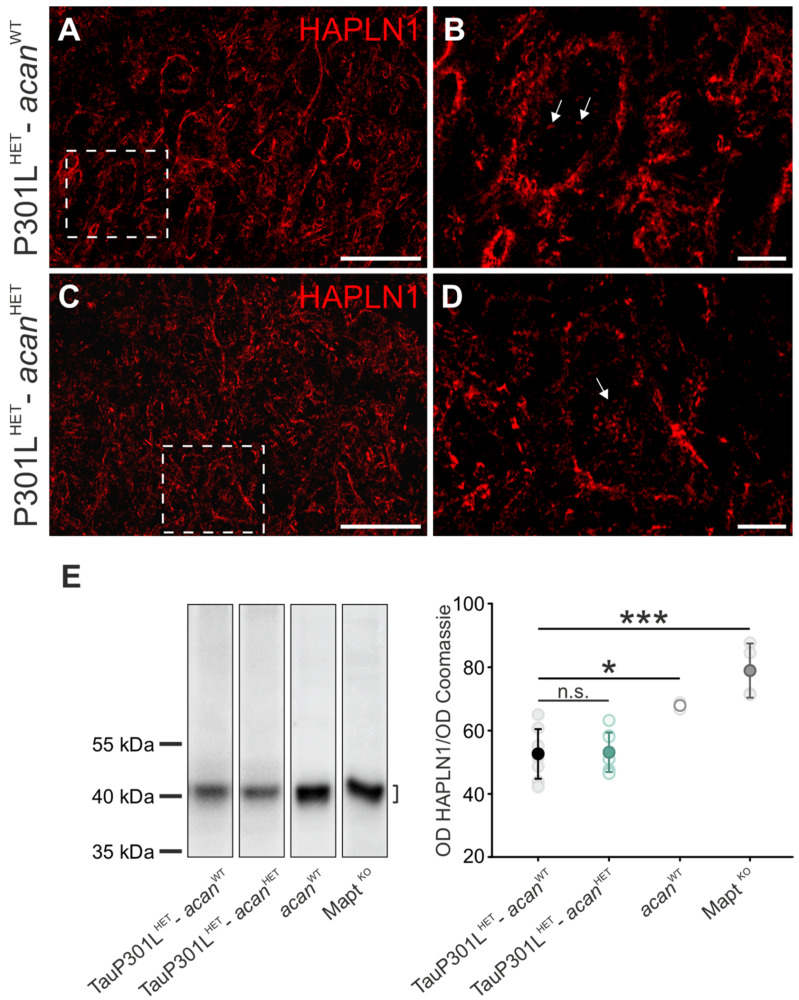
Characterization of HAPLN1 in the Mo5 of TauP301L-*acan* mice. (**A**–**D**) Immunohistochemical analysis of HAPLN1 (AF2608 (R&D Systems)) in the motor trigeminal nucleus (Mo5) of 3 TauP301L^HET^-*acan*^WT^ (**A**,**B**) and 3 TauP301L^HET^-*acan*^HET^ mice (**C**,**D**). High magnification confocal images (**B**,**D**) of HAPLN1 immunoreaction visualize location and structure of immunoreaction. Comparison of the structure of HAPLN1 immunoreaction revealed slight genotype-specific difference (**B**,**D**). HAPLN1 immunoreaction was mostly detected extracellularly in TauP301L^HET^-*acan*^HET^ (**C**) and TauP301L^HET^-*acan*^WT^ mice (**A**). Additionally, an intracellularly clustered HAPLN1 immunoreaction was detected in a few cells of both genotypes (**B**,**D**_,_ arrows). (**E**) Western blot analysis of HAPLN1 protein level (AF2608 (R&D Systems), at ~40 kDa) in the brainstem of TauP301L^HET^-*acan*^HET^ and TauP301L^HET^-*acan*^WT^ mice did not reveal any genotype-specific difference (*p* = 0.885, *n* = 8, *t*-test). HAPLN1 protein amount was significantly increased in *acan*^WT^ mice compared to TauP301L^HET^-*acan*^WT^ mice (*p* = 0.009, *acan*^WT^ *n* = 3, TauP301L^HET^-*acan*^WT^ *n* = 8, *t*-test). *Mapt ^KO^* mice revealed a significantly increased HAPLN1 protein level compared to TauP301L^HET^-*acan*^WT^ mice (*p* < 0.001, *Mapt ^KO^ n* = 4, TauP301L^HET^-*acan*^WT^ *n* = 8, *t*-test). For Western blot analysis, protein extracts from homogenized brainstems of 8 TauP301L^HET^-*acan*^WT^, 8 TauP301L^HET^-*acan*^HET^, 3 *acan*^WT^ and 4 *Mapt ^KO^* mice were used. Optical density (OD) of the specific chemoluminescence signal was quantified in relation to Coomassie stained total protein bands. Quantified areas on the Western blot lanes were marked by a bracket. For illustration of exemplary blot lanes, brightness and contrast were adapted. Data are given as mean ± SD, * *p* < 0.05, *** *p* < 0.001, n.s. not significant. Scale bar in (**A**,**C**) 50 µm, in (**B**,**D**) 10 µm.

Immunohistochemical analysis of HAPLN1 revealed slight structural differences in TauP301L^HET^-*acan*^WT^ (Figure 3A,B) and TauP301L^HET^-*acan*^HET^ mice (Figure 3C,D). While the extracellular HAPLN1 immunolabelling appeared fragmented in TauP301L^HET^-*acan*^HET^ mice (Figure 3D), it showed the typical enclosed loose appearance in TauP301L^HET^-*acan*^WT^ mice (Figure 3B). HAPLN1 immunoreaction was detected around cholinergic Mo5 motor neurons and in the neuropil in both genotypes (Figure 3A,C). In addition, an intracellularly clustered immunoreaction was detected in both TauP301L^HET^-*acan*^WT^ (Figure 3B, arrows) and TauP301L^HET^-*acan*^HET^ mice (Figure 3D, arrows).

Western blot analysis was performed to investigate HAPLN1 protein level (Figure 3E). The protein amount of HAPLN1 did not yield any genotype-specific difference in TauP301L^HET^-*acan*^HET^ and TauP301L^HET^-*acan*^WT^ mice (Figure 3E). Western blot analysis of HAPLN1 in *acan*^WT^ mice (67.84 ± 1, *n* = 3) and *Mapt ^KO^* mice (78.87 ± 8.54, *n* = 4) revealed a significantly increased protein level compared to TauP301L^HET^-*acan*^WT^ mice (52.55 ± 7.8, *n* = 8; *acan^WT^*: *p* = 0.009, *t*-test; *Mapt ^KO^ p* < 0.001, *t*-test, Figure 3E). The analysis of HAPLN1 protein level in BALB/c compared to C57BL/6 mice did not reveal any genotype-specific difference (data not shown). Thus, strain-specific differences in HAPLN1 protein level could be excluded.

#### 3.1.4. Hyaluronic Acid

**Figure 4:**
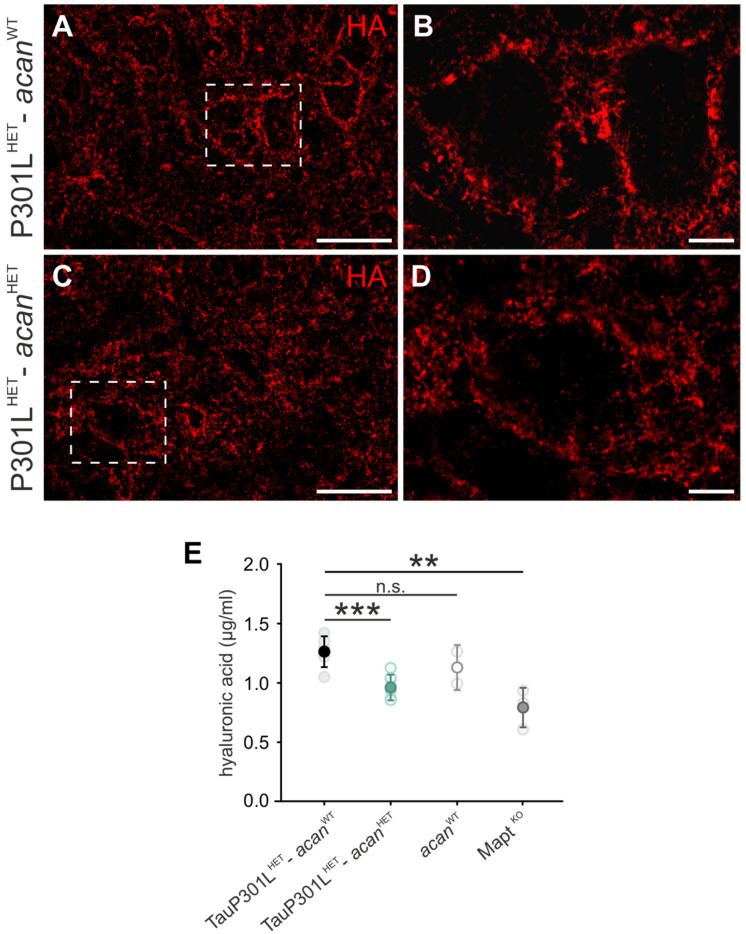
Characterization of hyaluronic acid in the Mo5 of TauP301-*acan* mice. (**A**–**D**) Immunohistochemical analysis of hyaluronic acid (HA) (bHABP (Merck Millipore)) in the motor trigeminal nucleus (Mo5) of 3 TauP301L^HET^-*acan*^WT^ (A, B) and 3 TauP301L^HET^-*acan*^HET^ mice (**C**,**D**). High magnification confocal images (**B**,**D**) of HA immunoreaction visualize location and structure of the immunoreaction. Comparison of the structure and location of HA immunoreaction did not reveal any genotype-specific differences between TauP301L^HET^-*acan*^WT^ (**B**) and TauP301L^HET^-*acan*^HET^ mice (**D**). Location of HA immunoreaction was limited to the extracellular area in both genotypes (**A**,**C**). (**E**) The amount of HA in TauP301L^HET^-*acan*^WT^ and TauP301L^HET^-*acan*^HET^ mice was determined by direct ELISA technique. The level of HA was significantly reduced in TauP301L^HET^-*acan*^HET^ mice compared to TauP301L^HET^-*acan*^WT^ mice (*p* < 0.001, TauP301L^HET^-*acan*^WT^ *n* = 6, TauP301L^HET^-*acan*^HET^ *n* = 7, *t*-test). Comparison of TauP301L^HET^-*acan*^WT^ and *acan^WT^* mice did not yield any genotype-specific difference. HA level was significantly decreased in in *Mapt ^KO^* mice compared to TauP301L^HET^-*acan*^WT^ mice (*p* = 0.002, TauP301L^HET^-*acan*^WT^ *n* = 3, *Mapt ^KO^ n* = 6, *t*-test). Data are given as mean ± SD, ** *p* < 0.01, *** *p* < 0.001, n.s. not significant. Scale bar in (**A**,**C**) 50 µm, in (**B**,**D**) 10 µm.

There were no obvious changes to the HA immunoreaction in TauP301L^HET^-*acan^WT^* mice (Figure 4A,B) and TauP301L^HET^-*acan^HET^* mice (Figure 4C,D). Both genotypes revealed an intense immunoreaction around cholinergic Mo5 motor neurons and in the neuropil (Figure 4A–D).

HA level was determined by ELISA technique and revealed a significant decrease in TauP301L^HET^-*acan^HET^* mice (0.956 ± 0.11, *n* = 7) compared to TauP301L^HET^-*acan^WT^* mice (1.258 ± 0.13, *n* = 6, *p* < 0.001, *t*-test, Figure 4E). The comparison of HA amount did not yield any genotype-specific difference in TauP301L^HET^-*acan^WT^* mice and *acan^WT^* mice (Figure 4E). Analysis of HA level revealed a significant decrease in *Mapt ^KO^* mice (0.787 ± 0.79, *n* = 3) compared to TauP301L^HET^-*acan^WT^* mice (1.258 ± 0.13, *n* = 6, *p* = 0.002, *t*-test, Figure 4E).

### 3.2. Characterization of Protein Levels of PP2A and PN Modulating Enzymes in Brains of TauP301L-acan Mice

Next, we investigated the molecular basis of the significant changes of protein levels of major PN components in TauP301L-*acan* mice. Based on earlier studies indicating that (i) PP2A dysregulation is related to the accumulation of pathological tau, which is a characteristic feature of tauopathies [70] and (ii) alterations in PP2A expression and activity modulate various ECM-associated proteolytic molecules [71], PP2A protein level was initially examined.

**Figure 5:**
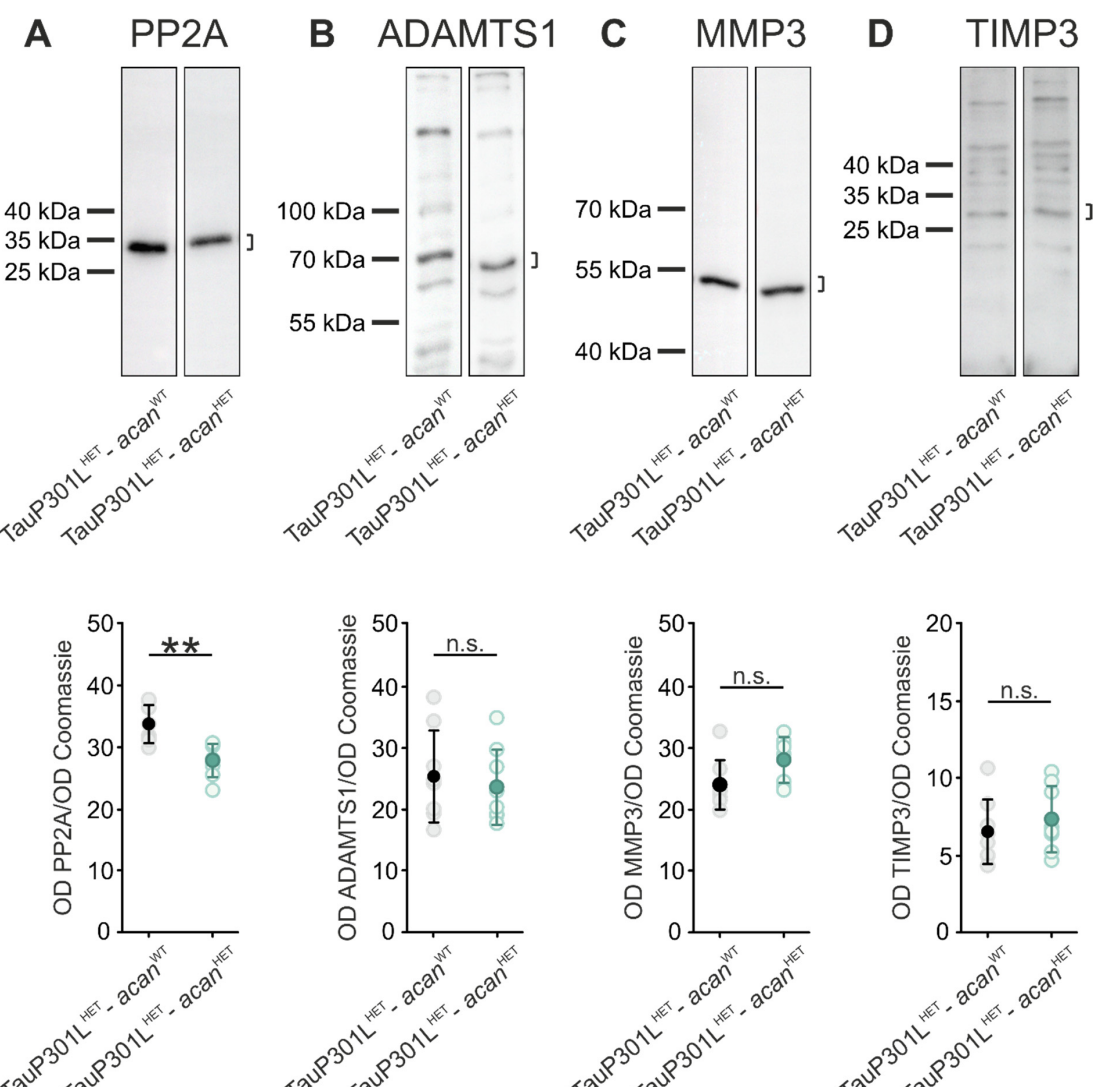
Protein level of PN modulating enzymes in the brainstem of TauP301-*acan* mice. (**A**) Western blot analysis of the amount of PP2A (clone E155 (Abcam), at ~35 kDa) in the brainstem with a significant decrease in TauP301L^HET^-*acan*^HET^ mice compared to TauP301L^HET^-*acan*^WT^ mice (*p* = 0.001, *n* = 8, *t*-test). (**B**–**D**) Western blot analysis of ADAMTS1 (B, AF5867 (R&D Systems), at ~70 kDA), MMP3 (C, clone EP1186Y (Abcam), at ~50 kDA) and TIMP3 (D, anti-TIMP3 (Proteintech), binds C-terminal, at ~30 kDa) in the brainstem of TauP301L^HET^-*acan*^HET^ and TauP301L^HET^-*acan*^WT^ mice did not reveal any genotype-specific difference (ADAMTS1: *p* = 0.620; MMP3: *p* = 0.055; TIMP3: *p* = 0.455, TauP301L^HET^-*acan*^WT^ *n* = 8, TauP301L^HET^-*acan*^HET^ *n* = 8, *t*-test). For Western blot analysis protein extracts from homogenized brainstems of 8 TauP301L^HET^-*acan*^WT^ and 8 TauP301L^HET^-*acan*^HET^ mice were used. Optical density (OD) of the specific chemoluminescence signal was quantified in relation to Coomassie stained total protein bands. Quantified areas on the Western blot lanes were marked by a bracket. For illustration of exemplary blot lanes, brightness and contrast were adapted. Data are given as mean ± SD, ** *p* < 0.01, n.s. not significant.

PP2A protein amount was determined by means of Western blot analysis and revealed a significant decreased PP2A protein level in TauP301L^HET^-*acan*^HET^ mice (27.78 ± 2.67, *n* = 8) compared to TauP301L^HET^-*acan*^WT^ mice (33.66 ± 3.07, *p* = 0.001, *n* = 8, *t*-test, Figure 5A).

Subsequently, protein levels of several proteolytic enzymes associated with continuous modification of perineuronal ECM and tau pathology [62,72,73,74,75,76] were determined by Western blot analysis. The comparison of ADAMTS1 (TauP301L^HET^-*acan*^WT^: 25.36 ± 7.46, TauP301L^HET^-*acan*^HET^: 23.62 ± 6.11, *p* = 0.62, *n* = 8, *t*-test, Figure 5B), MMP3 (TauP301L^HET^-*acan*^WT^: 23.83 ± 4.00, TauP301L^HET^-*acan*^HET^: 27.87 ± 3.72, *p* = 0.055, *n* = 8, *t*-test, Figure 5C) and TIMP3 (TauP301L^HET^-*acan*^WT^: 6.51 ± 2.1, TauP301L^HET^-*acan*^HET^: 7.32 ± 2.15, *p* = 0.46, *n* = 8, *t*-test, Figure 5D) protein level did not yield any genotype-specific difference in TauP301L^HET^-*acan*^WT^ and TauP301L^HET^-*acan*^HET^ mice (Figure 5B–D).

Thus, the present results lead to the conclusion that ADAMTS1, MMP3, and TIMP3 do not have a significant impact on the modified constitution of PNs in the brainstems of TauP301L-*acan* mice.

### 3.3. Co-Immunoprecipitation of Major Perineuronal Net Components and Tau Protein

Recent immunohistochemical investigations of TauP301L-*acan* mice showed an intracellularly clustered immunoreaction (Figure 1 and Figure 3) as well as an intracellular accumulation of tau in the Mo5 [40]. Thus, it is supposed that the intracellular accumulation of PN components is attributed to an interaction of PN molecules and tau, which prevents the secretion of intracellularly translated PN components.

**Figure 6:**
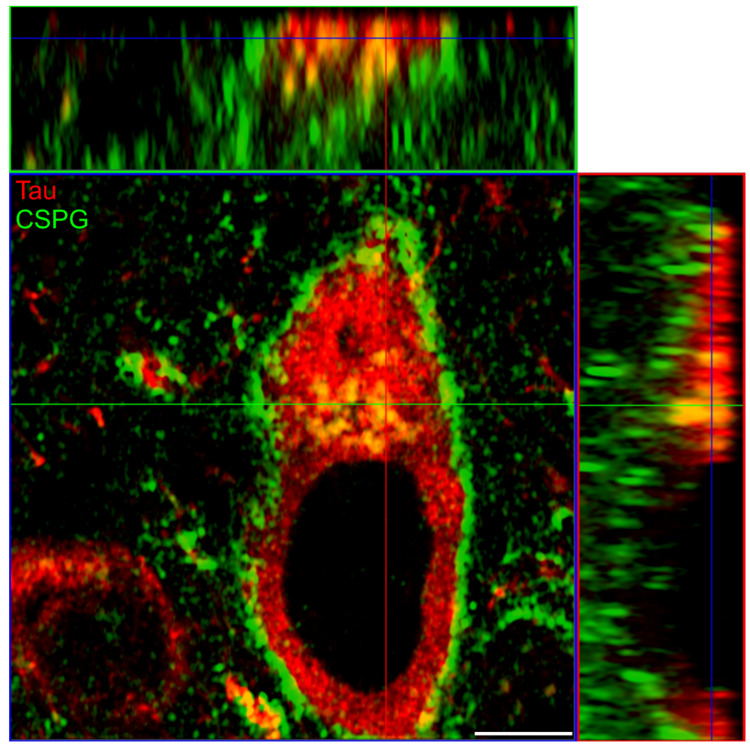
Location of tau protein and major PN components in the brainstem of TauP301L^HET^-*acan*^WT^ mice. Representative immunofluorescent image of human P301L tau visualized with anti-human total tau antibody (red, clone 7E5 (Roboscreen)) and PN-associated CSPGs using anti-brevican antibody (BCAN, green, clone 2 (BD Biosciences)). Orthogonal view of the high magnification confocal image revealed an intracellular colocalization of human P301L mutated tau (red) and the CSPG brevican in brainstem neurons. Scale bar in 5 µm.

To confirm this assumption, we first determined the location of human P301L tau and PN-associated CSPGs in TauP301L-*acan* mice by immunohistochemical analysis. High resolution confocal images demonstrated an intracellular colocalization of human P301L tau with PN-associated CSPGs in TauP301L-*acan* mice. (Figure 6).

**Figure 7:**
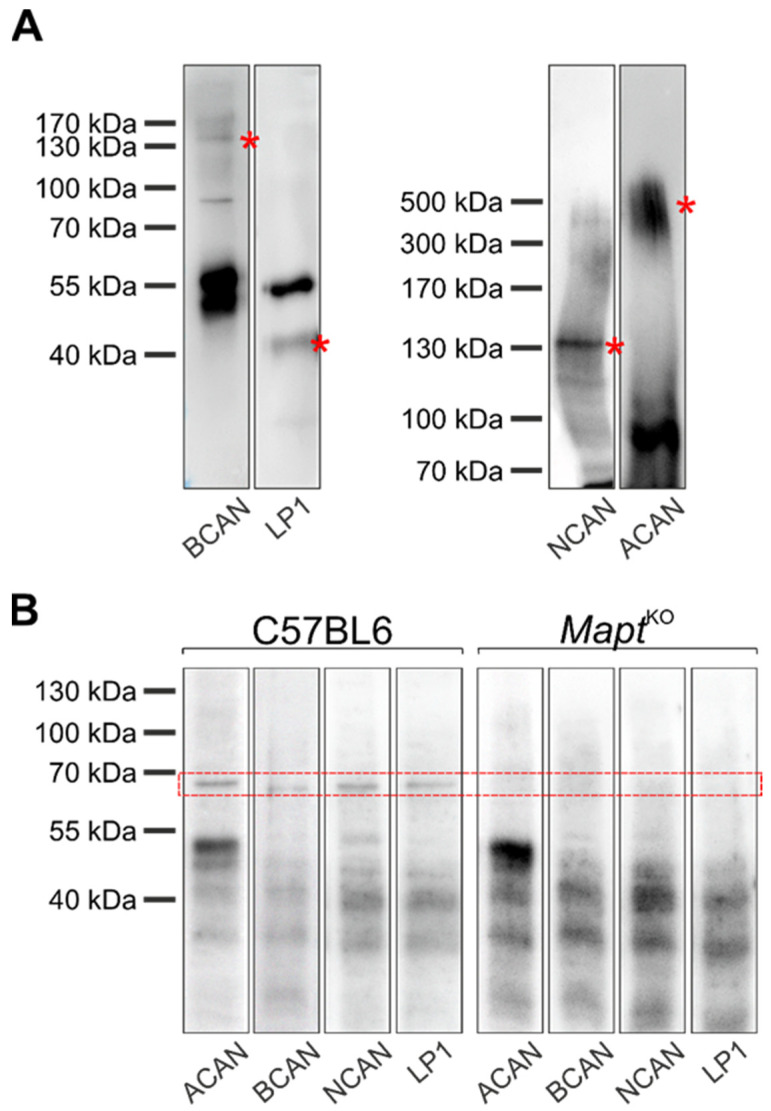
Interaction of perineuronal net components and tau protein. The interaction of several perineuronal net (PN) components and tau protein was investigated by combined co-immunoprecipitaion and Western blot analysis. (**A**) Verification of the target structure extraction (aggrecan (ACAN): AB1031 (Merck Millipore), at ~450 kDa; brevican (BCAN): clone 2 (BD Bioscience), at ~ 145 kDa; neurocan (NCAN): AF5800 (R&D Systems), at ~150 kDa; HAPLN1: AF2608 (R&D Systems), at ~40 kDa) by Dynabead-antibody-complex. Target structure specific bands were marked by an asterisk. The prominent signal about 55 kDa is attributed to the IgG heavy chain of the capture antibodies. (**B**) The formation of a PN-molecule-tau-complex was determined by total tau antibody (anti-pan tau (Dako)). A prominent tau-specific signal at ~65 kDa was detected when using C57BL6 brain homogenates for co-immunoprecipitation. When using brain homogenates of *Mapt ^KO^* mice, no tau-specific signal was detected. The band pattern below 55 kDa in C57BL6 and *Mapt ^KO^* mice is attributed to the IgG heavy and light chain of the applied capture antibodies. For illustration of exemplary blot lanes, brightness and contrast were adapted.

Subsequently, the potential interaction of PN molecules and tau was determined by means of co-immunoprecipitation in combination with Western blot analysis. First, the capture of the target structures (aggrecan ~450 kDa, brevican ~ 145 kDa, neurocan: ~150 kDa, HAPLN1: ~40 kDa) by Dynabead-antibody-complex was demonstrated (Figure 7A). Additionally, a prominent band about 55 kDa was detected (Figure 7A). This signal is most likely attributed to the immunoglobulin G (IgG) heavy chain of the used capture antibodies.

Next, it was analyzed whether a PN-molecule-tau-complex was formed. The investigation revealed an unambiguous interaction between several PN components (including aggrecan, brevican, neurocan, and HAPLN1) and tau protein, visualized by a prominent tau-specific signal at ~65 kDa (Figure 7B). The negative control (*Mapt ^KO^* mice) did not show a prominent tau signal at ~65 kDa (Figure 7B). The detected band pattern below 55 kDa is most likely related to the IgG heavy and light chain of the applied capture antibodies (Figure 7B).

These data demonstrate that major PN molecules and tau show intracellular protein interaction.

## 4. Discussion

The present work demonstrated that in a tau/aggrecan double transgenic mouse model expressing P301L tau with either aggrecan wildtype (two functional aggrecan alleles) or heterozygous background (one functional aggrecan allele) [40] PNs reveal significant modifications in the protein level of individual components accompanied by changes in the expression levels of PP2A, an enzyme involved in tau phosphorylation. We assume that this result is directly linked to the elevation of endogenous and transgenic human P301L tau protein levels in the TauP301L^HET^-*acan*^HET^ mice and hypothesize a complex, mutual interrelation of tau protein and PNs.

Previously, we generated this mouse model to gain more knowledge on the mechanisms underlying the neuroprotective features of aggrecan, which are poorly understood [40]. We especially focused on the contribution of aggrecan to intracellularly mediated processes that might trigger neuroprotection in addition to the extracellular guarding of neurons by PNs. The investigation of this mouse model did not reveal a direct protective effect of aggrecan on mutation-triggered intracellular accumulation of pathological tau aggregations [40]. However, the protein levels of endogenous and P301L mutated tau were significantly increased in TauP301L^HET^-*acan*^HET^ mice compared to TauP301L^HET^-*acan*^WT^ mice [40]. Thus, aggrecan seems to control intracellular mechanisms involved in the regulation of tau protein expression or turnover.

Neuropathological processes have been shown to be connected to physiologically relevant changes in PN structure and protein expression of PN components [46,77,78]. Aggrecan, as a major constituent of PNs, is supposed to be structurally and functionally dependent on the action of other PN components [40], and a central question in this study was to analyze the changes in the expression of major PN components linked to the variation in tau protein expression. There is increasing evidence that PNs are decisively involved in controlling plasticity, axonal growth, and regeneration as well as memory storage during development and in adulthood [32,79,80,81,82]. These factors play a crucial role in the progression of various neurological diseases, including different forms of tauopathies [82]. The backbone of PNs is composed of HA, which is synthesized by hyaluronan synthases (HASs) and is evenly distributed in the entire murine brain [83,84]. The present data indicate that increased levels of endogenous murine tau and transgenic human P301L tau in TauP301L^HET^-*acan*^HET^ mice [40] resulted in a decreased level of HA in this genotype. These findings might be explained by pathological tau-dependent changes in HASs expression and distribution, which result in an altered ratio of HA [84].

The investigation of perineuronal ECM related to tauopathies, especially in AD, revealed a significant increase in protein levels of major PN components [46,85,86]. The present study confirmed these findings by significant increased levels of brevican and neurocan in TauP301L^HET^-*acan*^HET^ mice. Additionally, the present data indicate that not only increased levels of dysfunctional tau protein but also the absence of tau protein in Mapt KO mice resulted in significant altered levels of PN molecules, including brevican, neurocan, and HAPLN1. This might be explained (i) by tau-dependent regulation of transcription and translation of PN components or (ii) by tau-dependent modulation of the ECM proteolytic system. With regard to the second point, tau protein might affect the complex interaction of matrix metalloproteinases (MMPs), a disintegrin and metalloproteinases (ADAMs), a disintegrin and metalloproteinases with thrombospondin motifs (ADAMTSs) and tissue inhibitors of metalloproteinases (TIMPs), which regulate the continuous remodeling processes of PNs [87,88,89,90]. In the present study we did not detect significant differences in MMP3, TIMP3, and ADAMTS1 protein level between TauP301L^HET^-*acan*^WT^ and TauP301L^HET^-*acan*^HET^ mice. As the analyzed proteins only represent a very small repertoire of PN-remodeling proteins, we still suspect that modifications of other subtypes of MMPs, ADAMs, ADAMTSs, and TIMPs contribute to the profound differences in PN structure and expression levels observed in TauP301L^HET^-*acan*^WT^ and TauP301L^HET^-*acan*^HET^ mice. Earlier studies have shown that alterations of the metalloproteinases (MPs) activity as well as an imbalance of MP-TIMP interactions are associated with neurodegenerative processes [91,92,93,94,95,96]. Investigation of *post-mortem* brains of AD patients showed that accumulations of MMPs and TIMPs were localized near Aβ-plaques and NFTs, thus MPs and their physiological inhibitors are assumed to be involved in the formation of these lesions [93,97]. For example, MMP9 and MMP3 levels were significantly increased in *post-mortem* brains of AD patients [72,98]. Mizoguchi and colleagues demonstrated that inhibition of MMP9 reduced neurotoxicity in Aβ-treated cell cultures and reversed Aβ-induced cognitive impairment in vivo [99]. Thus, it is suggested that MMP9 is a promising molecular target in AD treatment [100,101]. As MMP9 also plays a crucial role in PN formation and remodeling by regulating cleavage of major PN components [102,103,104], pharmacological modulation of MMP9 levels in AD therapy would also affect PNs, which could have far-reaching consequences for healthy brain function. Furthermore, it has also been shown that ADAMTS mRNA and protein levels are altered in AD. Thus, Sato and colleagues [105] demonstrated that ADAMTS4 mRNA is induced by Aβ-treatment, suggesting a crucial role in AD progression. Additionally, investigation on human *post-mortem* brains of AD patients yielded reduced protein amounts of ADAMTS4 and 5, and complete absence of ADAMTS9, pointing to a malfunction of PN component degradation [106].

Alterations in levels of various MMPs in cerebrospinal fluid (CSF) [107,108] have already been associated with AD. Furthermore, the simultaneous investigation of MMP9, MMP2, and TIMP1 in CSF might provide the potential to distinguish between different types of dementia [92,109].

In addition, studies in human *post-mortem* brains of AD patients demonstrated that the protein level of brevican as well as the amount of ADAMTS4-cleavage brevican fragments was significantly increased [47,85]. In the CSF, levels of brevican continuously decreased after traumatic brain injury and accomplished lower levels than patients with neurodegenerative diseases [110]. Based on these facts, Jonesco and colleagues [111] developed assays that are able to detect N-terminal and ADAMTS4-cleaved brevican in blood serum. Additionally, both markers revealed differential expression patterns between dementia patients and control subjects. However, the potential for using both assays as an early and reliable diagnostic tool is elusive [111] and thus, a more extensive analysis of the correlation between PN components and tau pathology is necessary.

Besides the intracellular processes of interrelated PN and tau modulation, PNs were also reported to act as an extracellular barrier against tau [112]. Pathological tau protein is not only generated intracellularly in affected cells but can also be released by neurons to the extracellular space and might be harmful to neighboring neurons [113,114,115,116,117,118]. Although ECM proteins are mainly transported in vesicles and tau is cytoplasmatically localized, the common meeting place might be the endocytosis of extracellular tau protein [119]. The extracellular accumulation is assumed to be one critical step in the global propagation of tauopathies [120,121]. PNs were shown to be of important relevance for inhibiting the diffusion of extracellular tau and its internalization into healthy neurons [38]. In the present study we hypothesized that these findings might be based on the capability of PNs to physically interact with tau. Indeed, co-immunoprecipitation experiments yielded physical connection between the main PN components and tau protein, indicating that the neuroprotective function of aggrecan and PNs in the extracellular space is based on the catching of tau and further prevention of cellular propagation.

In conclusion, the present data clearly demonstrate that aggrecan-based PNs and tau protein are functionally linked by mutual intracellular interferences, though, the relevance for neuroprotection in this context remains elusive. Still, there is increasing evidence showing that aggrecan and other PN components act as external shields protecting neurons from extracellularly propagating tau aggregates.

## Figures and Tables

**Table 1:** Antibodies used for detection of perineuronal net components, enzymes and tau protein forms. Immunohistochemistry—IHC; Western blot—WB; Immunoprecipitation—IP.

Detected Components	Dilution	Source	References/RRID
ADAMTS1 (AF5867)	WB 1:100	R&D Systems	[53]; RRID: AB_2044595
Aggrecan, core protein (AB1031)	IHC 1:1000	Merck Millipore	[54]; RRID: AB_90460
	WB 1:1000		
	IP 1:50		
Brevican, core protein (clone 2)	IHC 1:1250	BD Biosciences	[54]; RRID: AB_398212
	WB 1:1250		
	IP 1:20		
MMP3, C-terminal part (clone EP1186Y)	WB 1:4000	Abcam	[55]; RRID:AB_881243
Neurocan, N-terminal part (AF5800)	IHC 1:400	R&D Systems	[54]; RRID: AB_2149717
	WB 1:2500		
	IP 1:8		
HAPLN1 (AF2608)	IHC 1:400	R&D Systems	[54]; RRID: AB_2116135
	WB 1:750		
	IP 1:8		
Hyaluronic acid binding protein	IHC 1:100	Merck Millipore	[56]; RRID: AB_2861303
pan tau, C-terminal tau epitope	WB 1:4000	Dako	[57]; RRID: AB_10013724
PP2A alpha + beta antibody (clone E155)	WB 1:15000	Abcam	[58]; RRID:AB_777385
human tau total (clone 7E5)	IHC 1:750	Roboscreen	[40], 847-0102006301
TIMP3, C-terminal TIMP3 epitope	WB 1:800	Proteintech	[59]; RRID: AB_2204973

## Data Availability

The data presented in this study are available on request from the corresponding author.
